# Next-Generation Sequencing-Based Analysis of Clinical and Pathological Features of *PIK3CA*-Mutated Breast Cancer

**DOI:** 10.3390/diagnostics13182887

**Published:** 2023-09-08

**Authors:** Jolanta Smok-Kalwat, Grzegorz Chmielewski, Rafał Stando, Jacek Sadowski, Paweł Macek, Artur Kowalik, Ewelina Nowak-Ozimek, Stanisław Góźdź

**Affiliations:** 1 Department of Clinical Oncology, Holycross Cancer Center, 25-734 Kielce, Poland; jolantasmok1@gmail.com (J.S.-K.); stanislawgozdz1@gmail.com (S.G.); 2 Department of Radiation Oncology, Holycross Cancer Center, 25-734 Kielce, Poland; rafal.stando@onkol.kielce.pl (R.S.); jacek.sadowski@onkol.kielce.pl (J.S.); 3 Institute of Medical Sciences, Collegium Medicum, Jan Kochanowski University, 25-516 Kielce, Poland; 4 Department of Oncology, Institute of Health Sciences, Collegium Medicum, Jan Kochanowski University, 25-516 Kielce, Poland; pawel.macek@onkol.kielce.pl; 5 Department of Epidemiology and Cancer Control, Holycross Cancer Centre, 25-734 Kielce, Poland; 6 Department of Molecular Diagnostics, Holycross Cancer Centre, 25-734 Kielce, Poland; arturko@onkol.kielce.pl (A.K.); ewelinano@onkol.kielce.pl (E.N.-O.); 7 Division of Medical Biology, Institute of Biology, Jan Kochanowski University, 25-406 Kielce, Poland

**Keywords:** breast cancer, *PIK3CA*, next-generation sequencing, molecular diagnostics, overall survival, progression-free survival

## Abstract

Phosphatidylinositol-4,5-bisphosphate 3-kinase catalytic subunit alpha (*PIK3CA*) is a well-known oncogene with a high prevalence of mutation in breast cancer patients. The effect of the mutation is a deregulation in phosphatidylinositol 3-kinase-related pathways, and, consequently, in unrestricted cell growth and differentiation. With the advent of precision oncology, *PIK3CA* has emerged as a pivotal treatment target, culminating in the recent approval of alpelisib. Despite years of research on this genetic alteration, certain aspects of its influence on the prognosis of breast cancer remain ambiguous. The purpose of this analysis is to characterize the clinical picture of breast cancer patients with *PIK3CA* mutation in comparison to the *PIK3CA*-wild-type group. We examined 103 tumor samples from 100 breast cancer patients using a next-generation sequencing panel. Presence of the mutation was linked to an older age at diagnosis, a lower expression of Ki67 protein, a greater percentage of tumors expressing progesterone receptors, and a notably higher incidence of metastatic disease at presentation. No significant differences were identified in overall and progression-free survival between the two groups. Our findings enhance the understanding of how *PIK3CA* mutations shape the clinical and prognostic landscape for breast cancer patients.

## 1. Introduction

Breast cancer (BC) stands as the most frequently diagnosed malignancy globally. It is also the most common cause of death from cancer in females [1]. In 2020, Poland recorded 17,511 cases of BC, while projections suggest that the United States will see 300,590 new cases of invasive BC in 2023 [2,3]. Alarmingly, it is estimated that around 13% of women will face such a diagnosis within their lifetime [4,5]. Consequently, BC remains both a clinical and socioeconomic challenge, with the disease burden continually rising. Understanding the physiological, genetic, and molecular background of breast cancer has become crucial in the development of treatment methods that have transformed patients’ prognosis and survival. Beginning with estrogen deprivation therapy in the 1960s and through to anti-human epidermal growth factor receptor 2 (HER2) antibodies in the late 1990s, we are now witnessing the emergence of highly personalized therapies, where the effectiveness can be contingent upon a single genetic mutation [6,7,8,9]. 

One of such genes is phosphatidylinositol-4,5-bisphosphate 3-kinase catalytic subunit alpha (*PIK3CA*). First characterized by Carpenter et al. in 1990, its oncogenic properties were later identified in 1997 by Chang et al. [10,11]. It is estimated to be mutated in approximately 30% of BC cases, making it the second most frequent mutation after tumor protein 53 (TP53) [12,13]. Activating mutations of *PIK3CA* disrupt the phosphatidylinositol 3-kinase (PI3K) messenger pathways by producing a defective p110α subunit of PI3K. This leads to a cascade of events resulting in unregulated cellular growth, proliferation, differentiation, and survival [14,15,16]. Extensive research over the years has underscored the pivotal role of PI3K pathway alterations in the development of both cancerous and benign diseases. Remarkably, *PIK3CA* mutations have been found in all human cancers and are especially predominant in endometrial, breast, and bladder cancers, as well as Klippel–Trenaunay syndrome, which is a condition under the umbrella of *PIK3CA*-related overgrowth spectrum diseases [17,18,19,20]. The last few years have witnessed tremendous strides in the clinical application of this knowledge, culminating in 2019 with the approval of alpelisib for the treatment of *PIK3CA*-mutated hormone receptor (HR)-positive, HER2-negative advanced, or metastatic breast cancer. Such a diagnosis has historically been linked to an unfavorable prognosis for the patient, in spite of numerous advancements in therapeutic interventions over the decades [21,22]. As a selective PI3K inhibitor, the drug alpelisib targets and suppresses the aberrant pathway, thereby mitigating the rate of unrestrained cell proliferation [23,24].

Although *PIK3CA* has become a firmly established treatment target, controversies linger regarding the effect of the mutation on BC progression [25,26]. While this alteration has been associated with worse progression-free and overall survival, questions remain about the mutation’s heterogeneity [27,28,29,30]. In this paper, we explore the clinical landscape of 100 *PIK3CA*-positive breast cancer patients, as identified by next-generation sequencing. The aim of this analysis is to compare the baseline characteristics of patients’ and tumors’ features, overall survival, and progression-free survival between *PIK3CA*-mutated and *PIK3CA*-wild-type groups. 

## 2. Materials and Methods

**Figure 1:**
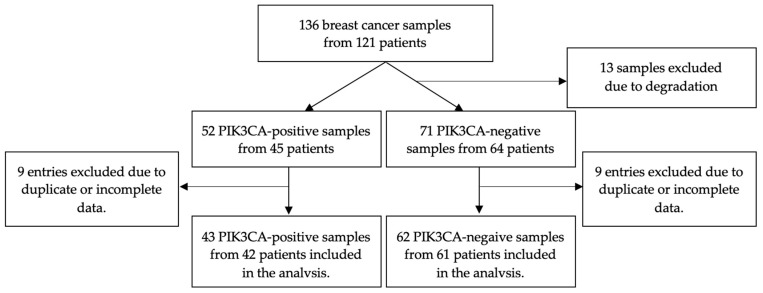
Flow diagram for sample inclusion in the analysis.

We evaluated 136 samples from 121 patients sourced from the Holycross Cancer Centre breast cancer archives (Figure 1). A total of 13 samples from 10 patients were excluded due to degradation. Eventually, after assessing the completeness of clinicopathological data, 103 samples from 100 patients were included. For patients diagnosed with two breast tumors, the cancers were classified as synchronous based on a maximum 6-month interval between diagnoses [31,32]. Bilateral breast cancer was differentiated from metastases to contralateral breast cancer based on histological type, grade, and absence of distant metastatic spread [33]. Our analysis encompassed demographic data, clinical and histopathological status, as well as follow-up findings. Patients included in the analysis were all Caucasian women aged 25 to 91 years and diagnosed between 2005 and 2022.

### 2.1. Genetic Analysis Methodology

#### 2.1.1. Library Preparation

Library preparations were conducted using the Ion AmpliSeq™ Cancer Hotspot Panel v.2 (CHPv2), Ion AmpliSeq Library Kit Plus, and Ion Xpress barcoded adapters (Thermo Fisher Scientific, Waltham, MA, USA) according to the manufacturer’s instructions (Thermo Fisher Scientific, Waltham, MA, USA). The panel covered hotspots of 50 tumor genes. However, only *PIK3CA* status was evaluated in this study, with 97 *PIK3CA* mutational hotspots analyzed. Multiplex PCR was conducted using a diluted 10 ng/μL genomic DNA with a premixed primer pool and Ion AmpliSeq HiFi master mix (Ion AmpliSeq Library Kit Plus, Thermo Fisher Scientific, Waltham, MA, USA). This was then followed by the partial digestion of the amplicons, where each sample was treated with FuPa reagent to partially digest the primer sequences and phosphorylate the amplicons. Following digestion, samples were briefly subjected to sequencing adapter ligation and were purified with the CleanNA reagent (CleanNA, Waddinxveen, the Netherlands). Post-purification, libraries were measured by quantitative PCR with real-time detection (qRT-PCR) using an Ion Library TaqMan™ Quantitation Kit (Thermo Fisher Scientific, Waltham, MA, USA) on the QuantStudio™ 5 Real-Time PCR System (Thermo Fisher Scientific, Waltham, MA, USA), adhering to the manufacturer’s protocols.

#### 2.1.2. Template Preparation and Sequencing

Libraries were normalized by dilution to 50 pM and either 16 or 32 libraries were pooled equimolarly. The clonal amplification of the barcoded DNA library (AmpliSeq libraries) onto ion spheres was performed using the Ion Chef™ instrument using Ion 520™ & Ion 530™ Kit-Chef according to the manufacturer’s instructions (Thermo Fisher Scientific, Waltham, MA, USA). The obtained barcoded libraries were multiplexed and loaded onto Ion 530™ Chips following the manufacturer’s protocol, and sequencing was executed on the Ion Gene Studio S5 system (Thermo Fisher Scientific, Waltham, MA, USA).

#### 2.1.3. Bioinformatic Analysis

The data from sequencing were processed using the Torrent Server Suite 5.12 (Thermo Fisher Scientific, Waltham, MA, USA). The sequencing data were aligned (mapped) to the reference sequence of the human genome (hg19). Samples were evaluated for genomic alterations, including single nucleotide variants (SNVs), as well as insertions and deletions, using the Variant Caller 5.12 program, which is part of the Torrent Server Suite 5.12. The default parameters used for CHPv2 data analysis were minimum allele frequency (SNP = 0.01/INDEL = 0.05, minimum quality = 10) and minimal sequencing depth = 10. Called variants were visualized using the Integrative Genomics Viewer (Broad Institute) (http://software.broadinstitute.org/software/igv/ accessed on 10 July 2023), which supports fast visualization of sophisticated variants. Coverage maps were generated using the coverageAnalysis plugin. The annotation of detected variants by the Torrent Server Suite 5.12 was performed with the wANNOVAR tool (http://wannovar.wglab.org/ accessed on 10 July 2023). Additionally, the Torrent Server Suite 5.12 was used to generate sequencing files in the FASTQ format. The FASTQ files were analyzed using the CLC Biomedical Genomics Workbench 5.0 (QIAGEN, Hilden, Germany) to eliminate erroneous base calling and to filter out potential strand-specific errors. The basic parameters used in the CLC analysis were as follows: minimum allele frequency = 0.01, minimal quality = 10, and minimal sequencing depth = 100. Detected variants were categorized based on the information from the ClinVar database and the American College of Medical Genetics and Genomics (ACMG) recommendations [1]. In addition, unclassified variants or conflicting results were analyzed using the in silico prediction tools in Varsome (https://varsome.com/ accessed on 10 July 2023), which integrates a literature search and valuable algorithms, population frequency, databases, etc.

### 2.2. Statistical Analysis

We evaluated associations between the presence of *PIK3CA* mutation as well as various clinical and histopathological findings using the t-test (or Mann–Whitney test in non-normal cases). Continuous data are presented as the mean (standard deviation) and median (lower/upper quartiles and ranges (minimum and maximum)). Categorical data are presented as numbers and percentages. The studied variables included age at diagnosis, histopathological grade, expression of estrogen and progesterone receptors (ERs, PRs), HER2 status based on immunohistochemistry (IHC) and fluorescent in situ hybridization (FISH) in IHC equivocal cases, Ki-67 status, tumor molecular subtype, clinical tumor, nodal status (pT and pN), clinical metastatic status (cM) clinical stage, and occurrence of relapse/metastatic spread or death. We also generated Kaplan–Meier curves for the overall survival (OS) and progression-free survival (PFS) in *PIK3CA*-mutated and *PIK3CA*-wild-type groups. The log-rank test was used to compare the OS and PFS in mutated and wild-type groups. Observations with a two-tailed p-value of less than 0.05 were considered “statistically significant”.

The starting point for the OS and PFS analyses was the histopathological confirmation of the diagnosis. The endpoint for OS was the patient’s death from any cause, and for PFS it was death from any cause, occurrence of metastasis, or recurrence of disease. Patients previously diagnosed with another neoplastic disease before BC were excluded from the OS and PFS analyses; this resulted in the exclusion of 4 patients—2 from the *PIK3CA*-WT group and 2 from the *PIK3CA*-M group. The statistical analysis was performed using the R software package, version 4.0.3.

## 3. Results

**Figure 2:**
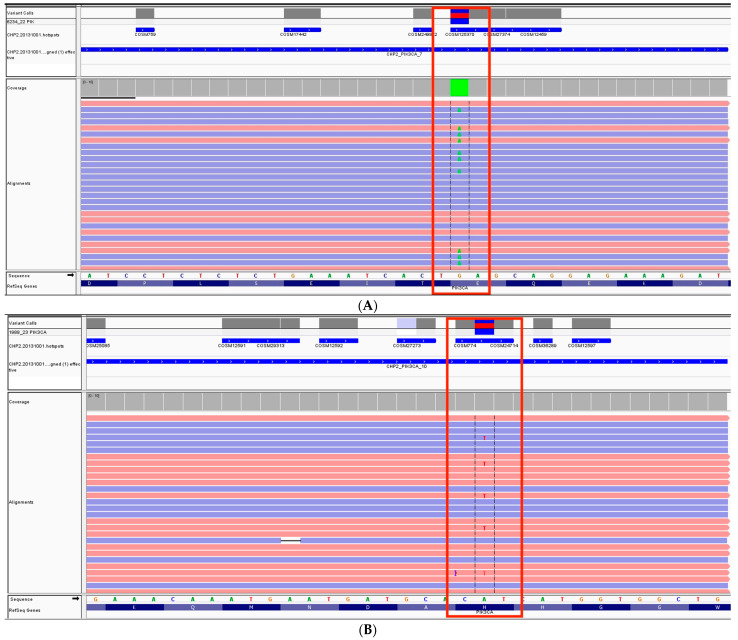
Examples of the detected mutations are highlighted in red (visualized using the Integrative Genomics Viewer): (**A**) c.1633G > A p. (Glu545Lys) in exon 10 of the *PIK3CA* gene. (**B**) c.3140A > T p. (His1047Leu) in exon 21 of the *PIK3CA* gene.

Of the 103 analyzed samples, *PIK3CA* mutations were found in 42 (41%) of them, whereas 61 (59%) were classified as *PIK3CA*-wild-type (*PIK3CA*-WT). These mutations were predominantly located in exons 10 and 21. Examples of mutations detected in those exons are presented in Figure 2. The mean age at diagnosis was 56.9 years ± 13.6 and was significantly higher in the *PIK3CA*-mutated (*PIK3CA*-M) group at 60.3 ± 14.0 compared to 54.6 ± 12.9 in the *PIK3CA*-wild-type group (OR: 1.45). The baseline characteristics of the studied population are presented in Table 1, while Table 2 displays the results of a univariate logistic regression analysis for the *PIK3CA*-M group.

None of the *PIK3CA*-M patients received alplelisib, attributed either to its unavailability or the lack of reimbursement by the Polish public health insurance at the time of treatment. Tumor characteristics indicate no notable differences in the histological grade or expression of estrogen receptors. However, progesterone receptor (PR) expression is significantly associated with the occurrence of *PIK3CA* mutation. PR expression was observed in 83.3% of *PIK3CA*-M tumors and 65.6% of *PIK3CA*-WT tumors (OR: 2.62). Average Ki-67 levels were significantly higher in *PIK3CA*-WT tumors (37.1% vs. 24.9%). We also classified Ki-67 values as “high” and “low” (applying a cutoff value of 20% and established for differentiating luminal A and luminal B tumors in our Center’s pathology department). With this approach, there was no statistically significant difference between the groups (OR: 0.45). Regarding molecular subtypes, luminal A and B tumors were the most prevalent in both groups. Triple-negative breast cancer (TNBC) constituted 8/61 (13.1%) of *PIK3CA*-WT tumors and only 2/42 (4.8%) of mutated tumors; however, this difference was not statistically significant.

In the pathological TNM staging, the T2 classification was dominant, followed by T4 in the *PIK3CA*-WT group and T3 in the *PIK3CA*-M patients. In terms of nodal classification, most of the patients in the *PIK3CA*-WT group were classified as N0 (57.9%), while in the mutated population there was an equal distribution between N0 and N1—both at 45.2%. We also classified the patients’ nodal status as N0 and “other than N0”, with the latter being 42.1% for the wild-type group and 54.8% for the mutated population (OR: 1.66). However, the results for T and N status did not show statistical significance. Interestingly, M1 status (and therefore, stage IV at initial diagnosis) was observed significantly more frequently in the mutated group (10/42, 23.8%, OR: 6.04) than in patients without the alteration (3/61, 4.9%).

Additionally, we focused on patients who were initially diagnosed with metastatic breast cancer (mBC), extracting this subgroup from the primary analyzed group. These insights are delineated in Table 3. Among the 13 tumors from patients with mBC, 10 (77%) had *PIK3CA* mutations. Bone metastases were the most common, followed by metastases to lung and liver. The average age at diagnosis of metastatic disease was 55 years for *PIK3CA*-WT patients and 62.4 years for *PIK3CA*-M patients—figures that align closely with the overall mean ages.

**Figure 3:**
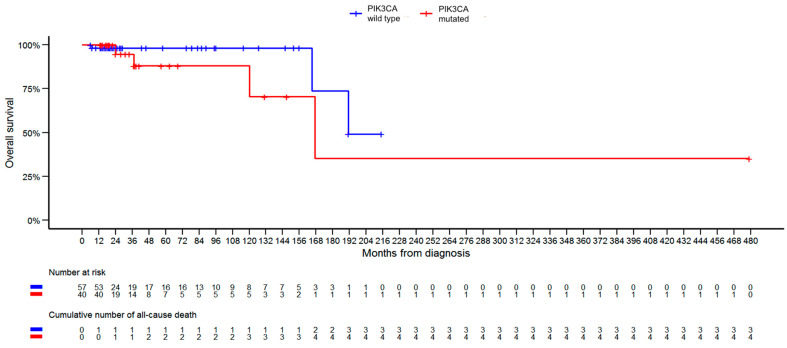
Kaplan–Meier overall survival curves for *PIK3CA*-mutated versus *PIK3CA*-wild-type patients. While the curves suggest a slight survival advantage for the *PIK3CA*-WT group, the difference is not statistically significant.

**Figure 4:**
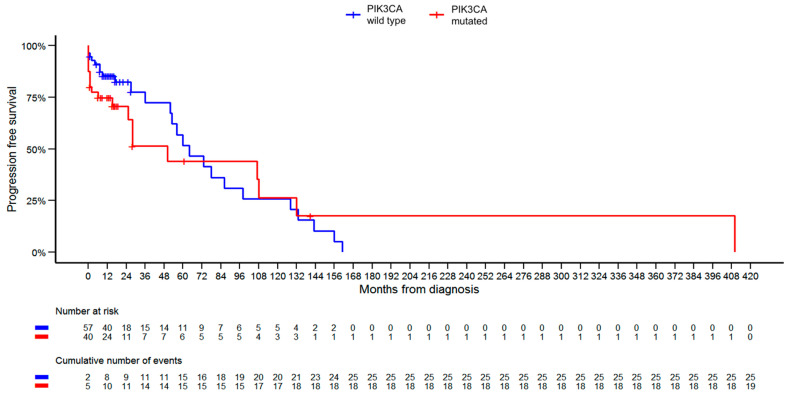
Kaplan–Meier progression-free survival curves for *PIK3CA*-mutated versus *PIK3CA*-wild -type patients. There is no significant difference between the curves.

Regarding patient survival within the studied group, a total of nine patients died: five from the *PIK3CA*-WT group and four from the *PIK3CA*-M group. Upon excluding patients diagnosed with two cancers from the survival evaluation, the total death count reduced to seven. The median OS for patients in the *PIK3CA*-WT group was 21.0 months, compared to 23.0 months in the *PIK3CA*-M group. The median PFS stood at 16.0 months for *PIK3CA*-WT patients and 14.0 months for those in the *PIK3CA*-M group. Our analysis revealed no statistically significant difference in OS and PFS rates between the groups. Kaplan–Meier curves for OS and PFS are presented in Figure 3 and Figure 4, respectively.

## 4. Discussion

First discovered in the early 1990s, the PI3K pathway has been extensively studied in cancer biology, with related mutations observed across all human cancers [34]. As this pathway is closely linked to the phosphatase and tensin homolog deleted on chromosome ten (PTEN) and Akt proteins, multiple mechanisms of tumorigenesis can arise from only a single mutation [35]. However, detailing the specific functional mechanisms of the pathway is beyond the scope of this paper. *PIK3CA* mutations have emerged as a point of interest in breast cancer diagnosis and treatment—especially since the advent of targeted therapies and the 2019 approval of alpelisib [21]. Our study explores the clinicopathological landscape of breast cancer patients who possess the *PIK3CA* mutation. With a 41% prevalence of *PIK3CA* mutation in our sample, the findings align with other research, considering that the median prevalence of this mutation in breast cancer patients is estimated to be 36% [36,37]. Our data indicate that patients with *PIK3CA* mutations are diagnosed at an older age, with a median age at diagnosis of 64 years compared to 55 years for those without the alteration. This older age at diagnosis in *PIK3CA*-M patients is consistent with the findings of other studies [38,39,40]. 

The expression of PR was significantly elevated in *PIK3CA*-M patients, which aligns with earlier findings [41,42]. The earliest reports linking *PIK3CA* alterations with higher expression of progesterone receptors date back to 2006, when Saal et al. investigated the relations between *PIK3CA*, PTEN loss, and hormone receptor status [43]. In our analysis, specimens with mutations had a reduced likelihood of being HER2-positive, although the difference was not statistically significant. This observation is in line with the existing literature [44,45]. The distribution of breast cancer subtypes showed no significant variation between the groups. A study by Reinhardt et al. demonstrated that *PIK3CA* mutations were significantly more prevalent in steroid hormone-receptor positive and HER2-negative tumors [46]. Similarly, Arsenic et al. reported that the most frequent subtype in *PIK3CA*-mutated patients was HR-positive and HER2-negative BC; however, their results were not statistically significant [29]. While analyzing the prevalence of TNBC, our results were not statistically significant. Nonetheless, the disparity between groups warrants further investigation. In our analysis, TNBC accounted for 13.1% of breast cancer subtypes in *PIK3CA*-M patients, which corresponds to the range reported in the literature, estimated at 5%–13.2% [47,48,49]. These findings reinforce the notion that *PIK3CA* is not a factor promoting triple negativity, as this percentage mirrors the overall proportion of TNBC in BC cases, estimated at 10–15% [50]. 

Interestingly, our analysis revealed a statistically significant difference in Ki67 levels, with lower expression in mutated tumors. Specifically, median expression stood at 17.5% in mutated samples, compared to 25% in wild-type samples. This implies a potentially less aggressive biology of the tumor, a conclusion that harmonizes with previous findings in the literature [41,51,52]. While both the heightened expression of PR and lower levels of Ki67 in *PIK3CA*-M tumors are described in the literature, the molecular background of this occurrence remains unexplained.

Alarmingly, the incidence of metastatic disease at the time of initial diagnosis was significantly higher in the *PIK3CA*-M group. Specifically, 23.8% of these patients presented with stage IV cancer, in contrast to 4.9% in the wild-type group. The prevalence of HR+/HER2− metastatic BC is well documented in the literature, and our findings corroborate these established observations. The seemingly paradoxical phenomenon, where a lower expression of Ki-67 and higher expression of PR coexists with a heightened metastatic potential, is unexplored in the current literature.

The introduction of alpelisib to the treatment of this specific patient group has received heightened research focus in recent years. The SOLAR-1 trial illustrated that the combination of alpelisib and fulvestrant is superior in terms of progression-free survival to placebo and fulvestrant [22,53]. Presently, the consensus in the literature is that *PIK3CA* mutations typically correlate with better OS. Yet, these mutations also seem to be linked to decreased chemotherapy sensitivity and poorer OS in stage IV disease [36,54,55,56]. Notably, our analysis diverges from these findings. While we did not observe significant differences in OS and PFS rates between the groups, the Kaplan–Meier curves demonstrate a slight advantage for the *PIK3CA*-WT group in terms of OS. A longer follow-up and a larger cohort would be necessary for validation of these findings.

There are some limitations of our analysis. Firstly, the sample size, while adequate to detect some significant trends, was relatively small. A larger study population would provide more statistical power and more robust insights. The analysis was performed retrospectively, and as such, some documentation associated with the diagnostic and treatment process was not complete or compliant with our requirements for inclusion in the analysis, which led to its exclusion. A prospective study protocol could have produced a more comprehensive dataset. It could also reduce the risk of selection bias, as only patients who underwent thorough genetic testing during their diagnostic process were included. Additionally, a multi-center study design could produce a more complex and nuanced understanding of any possible geographical trends we could not have observed in our single-center setting. 

On the other hand, there are also strengths in our approach. Unlike many recent studies, we included patients irrespective of their ER, PR, HER2, and cM status. This offers a broader look at the effects of the presence of the mutation. Another strength is the application of next-generation sequencing techniques, providing us with comprehensive coverage of the studied genome regions. 

## 5. Conclusions

*PIK3CA* remains a focal point for both molecular and clinical research, and our analysis adds to a growing body of knowledge surrounding it. Our findings affirm that the presence of this mutation in breast cancer patients is associated with distinct clinical presentation: older at diagnosis, higher prevalence of progesterone receptors in the tumor tissue, and, most notably, the patients are more commonly diagnosed with metastatic disease. Even though substantial progress has been made on the matter, there are still some unknowns [36]. Notably, our results challenge prior findings: in our analysis, OS Kaplan–Meier curves diverge, suggesting a potentially worse prognosis associated with the mutation on longer follow-up, regardless of the stage at diagnosis. Existing research suggests that the *PIK3CA* mutation may be a heterogenic entity, with prognosis dependent on a specific subtype of the mutation. The most commonly observed subtypes are H1047R, followed by E545K and E542K substitutions [12]. We anticipate that further research will delve deeper into this matter, aiming to provide a detailed breakdown of *PIK3CA* mutation subtypes and their clinical implications.

## Figures and Tables

**Table 1:** Baseline characteristics of the studied groups. Characteristics highlighted in red indicate a statistically significant difference between groups.

	*PIK3CA*-WT (*n* = 61)	*PIK3CA*-M (*n* = 42)	Total (*n* = 103)	*p*-Value
Age at diagnosis [years]				0.0338
Mean (SD)	54.6 (12.9)	60.3 (14.0)	56.9 (13.6)	
Median (Q1, Q3)	55.0 (44.0, 63.0)	64.0 (50.5, 70.0)	59.0 (48.0, 64.5)	
Range	25.0–83.0	25.0–91.0	25.0–91.0	
Grade				0.4901
Missing data	7	0	7	
G1	17 (31.5%)	15 (35.7%)	32 (33.3%)	
G2	24 (44.4%)	21 (50.0%)	45 (46.9%)	
G3	13 (24.1%)	6 (14.3%)	19 (19.8%)	
ER				0.1133
Negative (−)	13 (21.3%)	4 (9.5%)	17 (16.5%)	
Positive (+, ++, +++)	48 (78.7%)	38 (90.5%)	86 (83.5%)	
PR				0.0465
Negative (−)	21 (34.4%)	7 (16.7%)	28 (27.2%)	
Positive (+, ++, +++)	40 (65.6%)	35 (83.3%)	75 (72.8%)	
HER2 ^1^				0.1648
Negative	51 (83.6%)	39 (92.9%)	90 (87.4%)	
Positive	10 (16.4%)	3 (7.1%)	13 (12.6%)	
Ki67 [%]				0.0408
Missing data	2	0	2	
Mean (SD)	37.1 (32.4)	24.9 (26.8)	32.0 (30.7)	
Median (Q1, Q3)	25.0 (10.0, 70.0)	17.5 (5.0, 30.0)	20.0 (10.0, 50.0)	
Range	1.0–100.0	1.0–95.0	1.0–100.0	
Ki67 groups				0.0556
Missing data	2	0	2	
High (≥20%)	31 (52.5%)	14 (33.3%)	45 (44.6%)	
Low (<20%)	28 (47.5%)	28 (66.7%)	56 (55.4%)	
Molecular subtype ^2^				0.2349
Borderline	0 (0.0%)	1 (2.4%)	1 (1.0%)	
Luminal A	26 (42.6%)	23 (54.8%)	49 (47.6%)	
Luminal B, HER2-negative	19 (31.1%)	14 (33.3%)	33 (32.0%)	
Luminal B, HER2 positive	4 (6.6%)	0 (0.0%)	4 (3.9%)	
HER2-positive, non-luminal	4 (6.6%)	2 (4.8%)	6 (5.8%)	
TNBC	8 (13.1%)	2 (4.8%)	10 (9.7%)	
TNBC				0.1937
no	53 (86.9%)	40 (95.2%)	93 (90.3%)	
yes	8 (13.1%)	2 (4.8%)	10 (9.7%)	
cT				0.4094
Missing data	3	0	3	
T1	9 (15.5%)	3 (7.1%)	12 (12.0%)	
T2	27 (46.6%)	19 (45.2%)	46 (46.0%)	
T3	10 (17.2%)	12 (28.6%)	22 (22.0%)	
T4	12 (20.7%)	8 (19.0%)	20 (20.0%)	
cT				0.3326
Missing data	3	0	3	
T1 or T2	36 (62.1%)	22 (52.4%)	58 (58.0%)	
T3 or T4	22 (37.9%)	20 (47.6%)	42 (42.0%)	
cN				0.2126
Missing data	4	0	4	
N0	33 (57.9%)	19 (45.2%)	52 (52.5%)	
other than N0	24 (42.1%)	23 (54.8%)	47 (47.5%)	
cN				0.5883
Missing data	4	0	4	
N0	33 (57.9%)	19 (45.2%)	52 (52.5%)	
N1	19 (33.3%)	19 (45.2%)	38 (38.4%)	
N2	3 (5.3%)	2 (4.8%)	5 (5.1%)	
N3	2 (3.5%)	2 (4.8%)	4 (4.0%)	
cM				0.0046
M0	58 (95.1%)	32 (76.2%)	90 (87.4%)	
M1	3 (4.9%)	10 (23.8%)	13 (12.6%)	
Stage				0.0329
I	9 (14.8%)	3 (7.1%)	12 (11.7%)	
II	31 (50.8%)	20 (47.6%)	51 (49.5%)	
III	18 (29.5%)	9 (21.4%)	27 (26.2%)	
IV	3 (4.9%)	10 (23.8%)	13 (12.6%)	
Stage				0.2686
I or II	40 (65.6%)	23 (54.8%)	63 (61.2%)	
III or IV	21 (34.4%)	19 (45.2%)	40 (38.8%)	
Stage				0.0046
I–III	58 (95.1%)	32 (76.2%)	90 (87.4%)	
IV	3 (4.9%)	10 (23.8%)	13 (12.6%)	
OS [months]				0.7934
Mean (SD)	49.8 (54.0)	48.5 (77.5)	49.2 (64.3)	
Median (Q1, Q3)	21.0 (17.0, 76.0)	23.0 (15.5, 41.0)	21.0 (16.5, 51.5)	
Range	6.0–215.0	13.0–479.0	6.0–479.0	
PFS [months]				0.2591
Mean (SD)	32.7 (40.7)	34.0 (68.9)	33.2 (53.7)	
Median (Q1, Q3)	16.0 (9.0, 36.0)	14.0 (6.0, 28.0)	16.0 (7.0, 28.0)	
Range	0.0–161.0	0.0–410.0	0.0–410.0	
Death				1
no	56 (91.8%)	38 (90.5%)	94 (91.3%)	
yes	5 (8.2%)	4 (9.5%)	9 (8.7%)	

Abbreviations: WT: wild-type; M: mutated; TNBC: triple-negative breast cancer; OS: overall survival; PFS: progression-free survival; Missing data: number of entries for which data are unavailable; +, ++, +++: weakly positive, moderately positive, strongly positive ^1^ HER-2 positivity is determined by immunohistochemistry positivity, further confirmed by fluorescent in situ hybridization. ^2^ Molecular subtypes were defined based on estrogen receptors, progesterone receptors, HER-2, and Ki-67 expression.

**Table 2:** Univariate binary logistic regression analysis of *PIK3CA*-M patients.

		OR	95% CI	*p*–Value
Age	1) <50 years			
	2) ≥50 years	1.45	0.59–3.54	0.417
Grade	1) G1			
	2) G2 or G3	0.83	0.35–1.94	0.6627
ER	1) Negative (−)			
	2) Positive (+, ++, +++)	2.57	0.78–8.53	0.1223
PR	1) Negative (−)			
	2) Positive (+, ++, +++)	2.62	1–6.91	0.0508
HER2 ^1^	1) Negative (−)			
	2) Positive (+, ++, +++)	0.39	0.1–1.52	0.1762
Ki67	1) Low (<20%)			
	2) High (≥20%)	0.45	0.2–1.03	0.0575
TNBC	1) no			
	2) yes	0.33	0.07–1.65	0.1767
cT	1) T1 or T2			
	2) T3 or T4	1.49	0.67–3.33	0.3335
cN	1) N0			
	2) Other than N0	1.66	0.75–3.72	0.2139
cM	1) M0			
	**2) M1**	**6.04**	**1.55–23.55**	**0.0096**
Stage	I			
	II	1.94	0.47–8.03	0.3629
	III	1.5	0.32–6.94	0.604
	**IV**	**10**	**1.59–62.73**	**0.014**
Stage	1) I or II			
	2) III or IV	1.57	0.7–3.52	0.2698
Stage	1) I or II or III			
	**2) IV**	**6.04**	**1.55–23.55**	**0.0096**
Death	1) no			
	2) yes	1.18	0.3–4.68	0.8148

Abbreviations: M: mutated; TNBC: triple-negative breast cancer; +, ++, +++: weakly positive, moderately positive, strongly positive; OR: odds ratio; 95% CI: 95% confidence interval. ^1^ HER-2 positivity is defined as immunohistochemistry positivity confirmed by fluorescent in situ hybridization.

**Table 3:** Characteristics of patients diagnosed with metastatic breast cancer at presentation.

No.	*PIK3CA* Status	Age at Diagnosis	HER2 Status	Metastasis Sites
1	*PIK3CA*-WT	62	Negative	Bone
2	*PIK3CA*-WT	46	Negative	Bone, liver
3	*PIK3CA*-WT	57	Negative	Bone, lung
4	*PIK3CA*-M	70	Negative	Lung
5	*PIK3CA*-M	60	Negative	Bone, pleura, ovaries
6	*PIK3CA*-M	75	Negative	Lung, bone
7	*PIK3CA*-M	50	Negative	Bone
8	*PIK3CA*-M	38	Negative	Liver
9	*PIK3CA*-M	69	Positive	Lung, pleura
10	*PIK3CA*-M	57	Negative	Bone
11	*PIK3CA*-M	64	Negative	Bone
12	*PIK3CA*-M	70	Negative	Liver
13	*PIK3CA*-M	71	Negative	Bone

Abbreviations: WT: wild-type; M: mutated.

## Data Availability

All data created while preparing the article is included in the article body.
